# Supplementary material for: A ZTF-7/RPS-2 complex mediates the cold-warm response in C. elegans
Source: PLoS Genet. 2023 Feb 10;19(2):e1010628. doi: 10.1371/journal.pgen.1010628 (PMC9949642; doi:10.1371/journal.pgen.1010628)
Supplement: S1 Table — (DOCX) [file pgen.1010628.s001.docx]

**S1 Table. Strains used in the work.**

| Stain | Genotype |  |  |  |
| --- | --- | --- | --- | --- |
| N2 |  |  |  |  |
| SHG2103 | *3xflag::gfp::exos-1(ustIS112);rrp-8::mcherry(ustIS128)* |  |  |  |
| SHG2104 | *3xflag::gfp::exos-2(ustIS113);rrp-8::mcherry(ustIS128)* |  |  |  |
| SHG1690 | *3xflag::gfp::exos-10(ustIS114);rrp-8::mcherry(ustIS128)* |  |  |  |
| SHG1702 | *mCherry::dis-3(ustIS115);gfp::rrp-8(ustIS76)* |  |  |  |
| SHG680 | *3xflag::gfp::exos-1(ustIS112)* |  |  |  |
| SHG679 | *3xflag::gfp::exos-2(ustIS113)* |  |  |  |
| SHG1044 | *3xflag::gfp::exos-10(ustIS114)* |  |  |  |
| SHG1092 | *rrp-8::mCherry(ustIS128)* |  |  |  |
| SHG1660 | *rbd-1::mCherry(ustIS207)* |  |  |  |
| SHG904 | *nucl-1::gfp::3xflag(ustIS279)* |  |  |  |
| SHG893 | *3xflag::gfp::c27f2.4(ustIS97)* |  |  |  |
| SHG1257 | *3xflag::gfp::rpoa-2(ustIS116);fib-1::mCherry(ustIS140)* |  |  |  |
| SHG2271 | *asp-17(ust190);3xflag::gfp::exos-10(ustIS114)* |  |  |  |
| SHG2272 | *zip-10(ust192);3xflag::gfp::exos-10(ustIS114)* |  |  |  |
| SHG2085 | *glr-3(ust166);3xflag::gfp::exos-10(ustIS114)* |  |  |  |
| SHG1295 | *ztf-7(ust117);3xflag::gfp::exos-10(ustIS114)* |  |  |  |
| SHG1297 | *ztf-7(ust118);3xflag::gfp::exos-10(ustIS114)* |  |  |  |
| SHG1299 | *ztf-7(ust119);3xflag::gfp::exos-10(ustIS114)* |  |  |  |
| SHG1443 | *ztf-7(ust119);3xflag::gfp::exos-1(ustIS112)* |  |  |  |
| SHG1444 | *ztf-7(ust119);3xflag::gfp::exos-2(ustIS113)* |  |  |  |
| SHG1296 | *ztf-7(ust118)* |  |  |  |
| SHG1298 | *ztf-7(ust119)* |  |  |  |
| SHG2270 | *ztf-7(ust117);mCherry::ztf-7(ustIS115);3xflag::gfp::exos-10(ustIS114)* |  |  |  |
| SHG1445 | *ztf-7(ust118);mCherry::ztf-7(ustIS155)* |  |  |  |
| SHG1446 | *ztf-7(ust119);mCherry::ztf-7(ustIS155)* |  |  |  |
| YY178 | *eri-1(mg366);3xflag::gfp::nrde-3(ggIS1)* |  |  |  |
| SHG2182 | *ztf-7(ust118);eri-1(mg366);3xflag::gfp::nrde-3(ggIS1)* |  |  |  |
| SHG2183 | *ztf-7(ust119);eri-1(mg366);3xflag::gfp::nrde-3(ggIS1)* |  |  |  |
| SHG1441 | *ztf-7::gfp::3xflag(ustIS174)* |  |  |  |
| SHG2088 | *3xHA::rps-2(ustIS327)* |  |  |  |
| SHG2089 | *3xHA::rps-2(ustIS327);ztf-7::gfp::3xflag(ustIS174)* |  |  |  |
| SHG1560 | *ztf-7::gfp::3xflag(ustIS174);lmn-1::mCherry(ustIS144)* |  |  |  |
| SHG1886 | *3xflag::gfp::exos-10(D303N;E305Q)(ustIS251)* |  |  |  |
| SHG1887 | *3xflag::gfp::exos-10(EXO domain deletion)(ustIS252)* |  |  |  |
| SHG1888 | *3xflag::gfp::exos-10(HRDC domain deletion)(ustIS253)* |  |  |  |
| SHG2267 | *ztf-7(ust119);3xflag::gfp::exos-10(D303N;E305Q)(ustIS251)* |  |  |  |
| SHG2268 | *ztf-7(ust119);3xflag::gfp::exos-10(EXO domain deletion)(ustIS252)* |  |  |  |
| SHG2269 | *ztf-7(ust119);3xflag::gfp::exos-10(HRDC domain deletion)(ustIS253)* |  |  |  |
