# Supplementary material for: A ZTF-7/RPS-2 complex mediates the cold-warm response in C. elegans
Source: PLoS Genet. 2023 Feb 10;19(2):e1010628. doi: 10.1371/journal.pgen.1010628 (PMC9949642; doi:10.1371/journal.pgen.1010628)
Supplement: S2 Table — (DOCX) [file pgen.1010628.s002.docx]

**S2 Table. Sequences of sgRNAs for CRISPR/Cas9-mediated gene editing.**

*ztf-7::gfp* sgRNA#1 TTCGTTATACATAGTACATTCGG

*ztf-7::gfp* sgRNA#2 ATGTACTATGTATAACGAATAGG

*ztf-7::gfp* sgRNA#3 ACAACAATCTAATGATTGATAGG

*mCherry::ztf-7* sgRNA#1 GAAATCGCCGACTTGCGAGGAGG

*mCherry::ztf-7* sgRNA#2 GCAATGACTAACCGATTTTCGGG

*mCherry::ztf-7* sgRNA#3 TTCGGGATAATTGAGATGAGGGG

*ztf-7* sgRNA#1 ATCAATCCAATCGTAAGGACTGG

*ztf-7* sgRNA#2 ATGAGCTTGACTGTTGCTCGCGG

*ztf-7* sgRNA#3 AAGTAGGAATCGCCTTCGCTTGG

*glr-3* sgRNA#1 TCTTGCTTGATCCTCCACAATGG

*glr-3* sgRNA#2 GACAGGTGAAGACTCACACATGG

*glr-3* sgRNA#3 CAATTGGTTCGACTAAAGCATGG

*asp-17* sgRNA#1 CTGCAGAGCAGAAAGGAGCTAGG

*asp-17* sgRNA#2 GGGCAGCATGCTGAGTAATCAGG

*asp-17* sgRNA#3 TGTTGGCAGATCCAGTGTCAAGG

*zip-10* sgRNA#1 GCTGATGAGAATAGAGATTCAGG

*zip-10* sgRNA#2 CTGAATTGAGCAAGCATTGCTGG

*zip-10* sgRNA#3 AACATCTACCACATCCAGCTCGG
