# Supplementary material for: A ZTF-7/RPS-2 complex mediates the cold-warm response in C. elegans
Source: PLoS Genet. 2023 Feb 10;19(2):e1010628. doi: 10.1371/journal.pgen.1010628 (PMC9949642; doi:10.1371/journal.pgen.1010628)
Supplement: S3 Table — (DOCX) [file pgen.1010628.s003.docx]

**S3 Table. Sequences of quantitative real-time PCR primers.**

*eft-3* E2L F GTAAGGGATCTTTCAAGTACGC

*eft-3* E2L R CATCGATGATGGTGATGTAGTAC

*ztf-7* qrt F TCTGGTGGAAGCAAGAACAA

*ztf-7* qrt R CTGTCCAGTCCTTACGATTG

*rps-0* qrt F CGTATCGATCATCAGGCTGT

*rps-0* qrt R ATTGAGCGTTCTCCCTTGT

*rps-2* qrt F CAGAAGCAGACCACTGCCG

*rps-2* qrt R GATGGCGGTAGCAACTTC

*rps-12* qrt F CTGACGCAGGAGGAGATG

*rps-12* qrt R GTGAGCCTCACGCTTGTC

*nol-56* qrt F TTGAGGTTAAGGAGAAGGTCAT

*nol-56* qrt R GAGCATCCTCGTCAACCT

*fib-1* qrt F GCGGTGGTGATCGAGGTG

*fib-1* qrt R GATGTGGTTCGACAACGACAGT

*rrp-8* qrt F GCTGAGAAACGAGAGAAAGT

*rrp-8* qrt R GATTTCTTGTCAGCTGCTGC

*mtr-4* qrt F ATGCTCTTGTTCTTAAGGGATC

*mtr-4* qrt R GAGCGACATCAAGCGTATT

*T22H9.1* qrt F AGGTGGAATCGGAGATTCC

*T22H9.1* qrt R GCAGCTTCAGCATCTTCCT

*M28.5* qrt F ACAGAGGAATCTCTGAGATCAT

*M28.5* qrt R GACGAGTGACTCCACAAGC
