# Supplementary material for: A ZTF-7/RPS-2 complex mediates the cold-warm response in C. elegans
Source: PLoS Genet. 2023 Feb 10;19(2):e1010628. doi: 10.1371/journal.pgen.1010628 (PMC9949642; doi:10.1371/journal.pgen.1010628)
Supplement: Attachment — Submitted filename: response_20230102.docx [file pgen.1010628.s004.docx]

Dear Dr. Ma,

Thank you very much for reviewing our manuscript. The reviewers have given us great and very helpful suggestions. We are very glad that they like our work and really appreciate their suggestions.

We have carefully revised the manuscript as the editor and reviewers suggested. Basically, we have added a number of new experiments and analysis:

(1) Included fluorescent density analysis of GFP::EXOS-10 in different mutant background (new Fig. 3B).
(2) conducted lifespan experiments of N2 and *ztf-7* mutants at 25℃ (new Fig. 3I).
(3) Assayed cold survival rate of N2 and *ztf-7* mutants (new Fig. 3J).
(4) qRT-PCR quantified the mRNA levels of *fib-1*, *nol-56*, *mtr-4*, *rrp-8*, *rps-2*, M28.5 and T22H9.1 after partial knocking down by RNAi for 12 hours (new Fig. 5E).
(5) Box plots showed relative fluorescent intensities of ZTF-7::GFP upon knocking down *rpl-2*, *rpl-19*, *rpl-30*, *rps-0*, *rps-1*, *rps-2*, *rps-4*, *rps-5*, *rps-9*, *rps-11* and *rps-12* (new Fig. 6E and Fig. 6I).
(6) qRT-PCR quantified the mRNA levels of *ztf-7* after feeding RNAi targeting *rps-2* (new Fig. 6F)*.*
(7) qRT-PCR quantified the mRNA levels of *rps-0*, *rps-2* and *rps-12* after partial knocking down by RNAi for 12 hours (new Fig. 7F).

In this revision, we have 7 main figures, and 3 supplementary tables. The revised texts are highlighted in red.

If there are any other questions or concerns, please let us know.

Thanks very much for the help.

Sincerely,

Shouhong

*Reviewer #1:*

*The authors demonstrate that ZTF-7 is required for RNA exosome depletion from nucleoli upon transient cold-warm exposure in C. elegans. These effects are dependent upon RPS-2, a ribosomal protein of the small subunit. These results establish a novel mechanism by which C. elegans responds to environmental cold-warm exposure. The concept of their work is of broad interest and the data they present are* *appealing. However, at the current state, the study has some weak points that limit its significance and need to be further investigated.

Major comments:
1. What mechanisms do these authors propose for the interaction of RPS-2 and ZTF-7 interaction? It should be noted that ZTF-7 is located* *predominantly in the* *cytoplasma. The fact that RPS-2 could interact with ZTF-7 implicates that RPS-2 is also located in the cytoplasma.*

RESPONSE: Thanks very much for the comments.

The interaction between RPS-2 and ZTF-7 are two folds. First, ZTF-7 associates with RPS-2 via protein-protein interaction. Second, RPS-2 likely promotes the transcription of *ztf-7* (new Fig. 6F). Yet the mechanism is still unclear.

RPS-2 is a component of 40S ribosomal subunit and therefore theoretically could locate in both nucleus for ribosome assembly and cytoplasm for translation at least. We attempted to generate RPS-2::GFP::3xFLAG transgene *in situ* by CRISPR/Cas9 technology. Unfortunately, homozygous RPS-2::GFP::3xFLAG transgenic animals were lethal, which indicated the GFP::3xFLAG tag impaired the function of RPS-2.

We have revised the discussion section to include the information (Lines 360-363).

*2. Knockdown of rps-2 by RNAi induced a significant decrease in ZTF-7 expression. These authors should clarify how RPS-2 regulates the expression of ZTF-7. This regulation occurs at the transcriptional or post-transcriptional level? The authors need to determine the mRNA expression of ztf-7 in worms subjected to rps-2 RNAi. Alternatively, interaction of RPS-2 with ZTF-7 probably promotes ZTF-7 protein stability.*

RESPONSE: Thanks very much for the suggestion.

We performed qRT-PCR to quantify mRNA levels of *ztf-7* after feeding RNAi targeting *rps-2* and found the mRNA level of *ztf-7* decreased to roughly 70% of that of control animals upon *rps-2* knockdown via RNAi (new Fig. 6F). We speculated that RPS-2 likely promotes the transcription of *ztf-7*, yet the mechanism is unclear*.*

We have revised the text to include the information (Lines 302-305).

*3. What is the physiological significance of cold-warm exposure-induced exosome translocation from the nucleolus to the nucleoplasm in this tissue? Does blockage of this exosome translocation influence life-history traits in worms.*

RESPONSE: Thanks very much for the comments.

To test whether ZTF-7 is involved in cold tolerance response, we grew L3-stage N2 and *ztf-7* animals at 3℃ for 48 hours and transferred back to 20℃ for 8 hours. Both *ztf-7 (ust118)* and *ztf-7 (ust119)* mutants exhibited higher survival rate than N2 animals after cold-warming stress (new Fig. 3J), suggesting that ZTF-7 limits the ability of animals to tolerate cold environment or cold-warm stress.

The results section was revised accordingly (lines 233-237).

Exosome translocated from the nucleolus to nucleoplasm upon cold-warm exposure, and restored the nucleolar accumulation during further recovery. The function of this exosome translocation is unclear. We speculated that cold-warm exposure may result in the accumulation of erroneous RNAs in certain subnuclear compartments and induced the translocation. Further investigations are required to test whether forced mislocalization of exosome factors could trigger distinct biological responses.

We have revised the discussion section to include the information (lines 350-355).

*4. The experiments in figure 2E and 2F lack important controls. It is hard to understand the importance of these results in figure 2. If the authors do not have additional comments that would highlight their significance, they should be transferred to a supplemental figure.*

RESPONSE: Thanks very much for the suggestion. We prefer to keep Figs. 2E-F in main figures.

GLR-3 encodes a kainate-type glutamate receptor and has been reported to sense cold in the peripheral sensory neuron ASER. ZIP-10 and ASP-17 mediate the cold-warm response and promote survival ability under resource-limiting and thermal stress conditions. Figures 2E-F showed that these known genes are not involved in the cold-warm shock induced exosome translocation response, suggesting that there exist additional pathways, other than GLR-3 or ASP-17/ZIP-10 axis, to response to cold environment or cold-warm stress. Therefore, we conducted the forward genetic screening and identified the ZTF-7/RPS-2 complex that represents a new cold-warm shock responsive pathway.

We have revised the results section to highlight the significance (lines 206-208).

*Minor issues:
1. Line 648, “C27F2.4” should be “C27F2.4::GFP”.*

RESPONSE: corrected.

*2. What type of tissue are these images taken from?*

RESPONSE: All the pictures were taken from epidermal cells. We have revised the materials and methods section (lines 537-538) and figure legends to include this information.

*3. The quantification of data in figure 6D-F is lacking.*

RESPONSE: Thanks very much for the comments.

The fluorescent intensity was quantified by ImageJ and shown in new Fig. 6E and 6I, respectively. In each group, at least 10 animals were measured.

For quantification of Western blotting, the band density was quantified by ImageJ and the values were included in new Fig. 6G.

The figure legends were also revised.

*4. Which band corresponding to* *ZTF-7::GFP::3xFLAG in figure 6B and 6C?*

RESPONSE: In Fig. 6B, both bands in the box are ZTF-7::GFP::3xFLAG. We had cut out both bands from the gel separately, subjected them to mass spectrometry, and both identified ZTF-7. In Fig. 6C, the input sample revealed that the both bands are ZTF-7::GFP::3xFLAG. The figure 6B legend was revised to include the information.

These data implied that ZTF-7 may have two isoforms, although the detailed sequences are unclear right now.

*5. There are many grammar errors in this manuscript.*

RESPONSE: Sorry for the mistakes. We have carefully examined the text and tried to fix all errors.

*Reviewer #2: In this highly interesting paper Xu el al. discover that cold-warm exposure translocates the exosome from the nucleolus to the nucleoplasm. They demonstrate this using a number of different and convincing experiments, and also have the proper controls to make sure this effect is specific to some nuceolus components but not others. This is especially interesting in light of their previous seminal work on risiRNAs and their links to ribosomal RNA processing and regulation. Then, using an unbiased genetic screen, they identify a conserved ZTF-7/RPS-2 complex which controls this cold-warm response. They validated the results of their screen and the target that they identified and proceed to biochemical analysis of the protein finding the relevant proteins with which it co-localizes and interacts. KD of these co-binding proteins cancels cold-warm controlled exosome translocation. In summary, using very well done experiments they find a novel and physiologically relevant cold response mechanism in animals. I don’t have any suggestions for additional experiments, in my opinion the paper is very interesting and would generate follow up studies in its current form.*

RESPONSE：Thanks very much for the comments. We are very glad that the reviewer likes our work.

*Reviewer #3: This manuscript by Xu, et al. explores the response to transient cold exposure followed by return to the preferred growth temperature (cold-warm shock) in the well-studied genetic model system C. elegans. They report that the sub-nuclear localization of RNA exosome subunits changes in response to cold-warm shock. This shift requires the primarily cytoplasmic protein ZTF-7 and its interactor RPS-2, and may define a new response pathway.

Overall, this manuscript is very well-written and the science was rigorous and convincing – a pleasure to read! There are a few places where the story could benefit from some additional clarity:

1. Figure 1c and many other graphs show the “percentage of nucleolar localization” on the y axis. Does this mean percent of nuclei with any nucleolar localization or does it indicate what fraction of the protein is in the nucleolus for any given nucleus (on average)? Presumably the former, but please clarify this.*

RESPONSE: Thanks for asking. Nucleolar localization is scored as positive if the fluorescent protein showed nucleolar enrichment in more than half of [epidermal](javascript:;) cells in one animal. In each group, more than 20 animals were counted. The figure 1C legends and materials and methods section (lines 537-540) have been revised to include the information.

*2. Figure 3b shows that for all three alleles of ztf-7 the exos-10 is not depleted from the nucleolus by cold-warm shock.* *The control nuclei at 20C show a visually apparent enrichment of exos-10 in the nucleolus. The ust119 nuclei appear to as well, but for ust117 and ust118, the fluorescence looks uniform. Is this just how these particular images appear, or is this a real difference?* *The line graphs of fluorescence intensity across the diameter of a nucleus as shown in Fig 1b, d, and f would be interesting to clarify this.*

RESPONSE: Thanks very much for the comments.

We re-took the photos and measured the fluorescence intensity across the diameter of a representative nucleus, as shown in new Fig. 3B. The nucleolar enrichment of EXOS-10 is similar in all three alleles of *ztf-7*.

*3. Figure 3h shows a normal lifespan of ztf-7 at 20C. Most published lifespans are performed at 25C – is the ztf-7 lifespan wild type at 25C as well?*

RESPONSE: Thanks very much for the suggestion.

We have included a new experiment and assayed the life span of *ztf-7* at 25℃ (new Fig. 3I). The *ztf-7* mutation did not pronouncedly affect the life span of animals at both 20°C and 25^o^C (Figs. 3H-I).

*4. Please clarify whether the nuclei in Figure 5a are from worms grown at 20C with no cold-warm shock.*

RESPONSE: Thanks for the reminding.

In Fig. 5A, the nuclei were from worms grown at 20℃ without cold-warm shock. The manuscript and figure legend have been revised to include the information (line 266 and legends).

*5. Are all cold-warm shock images taken at the 0.5 hour recovery timepoint – e.g. Fig 5c? Please clarify in the figure legend or elsewhere.*

RESPONSE: Thanks very much for the comments.

All cold-warm shock images were taken at the 0.5 hour recovery time point unless otherwise specified (lines 184-185). The figure legends have also been revised to include the information in Figs. 5C, 7B-E.

*6. When partial RNAi depletion is done,* *it is typical to measure mRNA levels by qPCR to assess how strong the knock down is. Can this be done for the partial RNAi experiments in this paper, e.g. Figure 5c?*

RESPONSE: Thanks very much for the suggestion.

We have included new experiments to measure mRNA levels of a number of rRNA processing factors after RNAi by qPCR (new Figs. 5E). Additionally, we also quantified the mRNA levels of *rps-0*, *rps-2* and *rps-12* after RNAi for 12 hours (new Fig. 7F). The depletion of these genes for 12 hours significantly blocked cold-warm shock-induced EXOS-10 translocation.

*7.* *Figure 6c is a little confusing since the IP-western is not labeled but doesn’t align exactly with* *the western of the input. Please either label both westerns or adjust the figure so that the blot slices are the same size and alignment.*

RESPONSE: Thanks very much for the suggestion. We have revised and reorganized the images in Fig 6C.

*8. What cell type are we looking at throughout this manuscript?*

RESPONSE: Sorry for the missing. All the pictures were taken from epidermic cells. We have revised the materials and methods section (lines 537-538) and figure legends to include this information.

*9. Please include statistical analyses, when appropriate. The methods section says a Student’s T-test or unpaired Wilcoxon test was done as indicated, but this is not indicated anywhere in the manuscript. Which data were analyzed statistically? Only Figure 3f and g? What test was done? This should be indicated in the figure legend. At minimum, figures 5c, 7b, 7c, 7d, and 7e should also have a statistical analysis.*

RESPONSE: Thanks very much for the suggestion.

We have revised the figure legends to include statistical analysis method and also marked in figures in Figs. 5C and 7B-E.
